# THE NATIONAL COLLABORATORY TO ADDRESS ELDER MISTREATMENT MENTORSHIP PROGRAM: KEY LEARNINGS AND OPPORTUNITIES

**DOI:** 10.1093/geroni/igae098.0264

**Published:** 2024-12-31

**Authors:** Lauren Bangerter, Kristin Lees Haggerty, Laura Mosqueda

**Affiliations:** Chair; Co-Chair; Discussant

## Abstract

The National Collaboratory to Address Elder Mistreatment (NCAEM) recognizes that improving elder mistreatment identification, intervention, and prevention requires diverse mentorship. The NCAEM Mentorship program is a one-year program designed to mentor clinicians, service providers, researchers, policymakers, and advocates who are focusing on improving care for older adults experiencing or at risk of experiencing elder mistreatment. This symposium will begin by an overview of the NCAEM mentoring program history and goals and presentations from current and past mentees. Dr. Troutman-Jordan’s presentation will discuss their mentee project that included focus groups with home health nurses, as well as the process of then serving as a mentor and highlight opportunities for collaborative mentoring to address elder abuse. Dr. Simley will describe a mentee project focused on developing a survey for the Parkinson’s Foundation to help generate data to inform the development of a continuing education module on elder mistreatment among individuals living with Parkinson’s disease. Dr. Roberts will will focus on the role of the NCAEM in catalyzing interdisciplinary elder abuse research in long-term care settings, including nursing homes and assisted living. Dr. Steinman’s presentation will describe a mentee project focused on the conceptualization and development of a national study of racial/ethnic group differences in older adults’ involvement in Adult Protective Services. Dr. Marrs’s presentation will focus on the links between ageism and elder abuse and describe the development of an ageism public awareness project funded by the Administration for Community Living. Our discussant, Dr. Mosqueda, will provide summary remarks and moderate discussion.

